# Supplementary material for: Drug repurposing for aging research using model organisms
Source: Aging Cell. 2017 Jun 16;16(5):1006–15. doi: 10.1111/acel.12626 (PMC5595691; doi:10.1111/acel.12626)
Supplement: Supplementary file 7 — Data S1 Zip‐Archive of all report cards. [file ACEL-16-1006-s007.zip › RC_CAQ.pdf]

CAQ

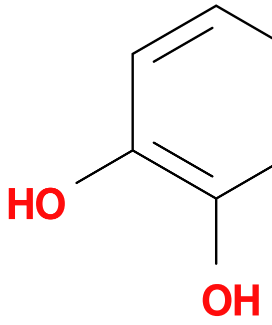

Database identifiers

ChEMBLCompound	CHEMBL280998
DrugBank	DB02232
CHEBI	18135
ZINC	ZINC13512214
eMolecules	480934

Ranking

	Rank	Score
Drosophila	422/697	0.339
C. elegans	NA	NA

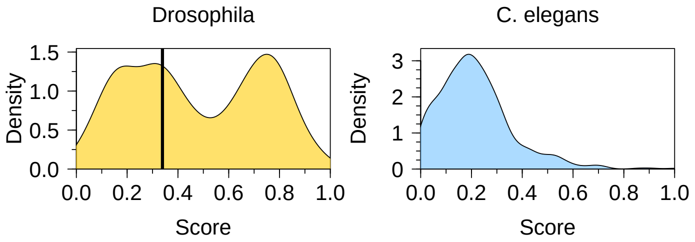

	Ageing implication	Domain conservation	Binding site conservation	Binding affinity	Bioavailability	Lipinski	Promiscuity	Purchasability	Drug approval	**Total**
Drosophila	1.0	0.917	0.966	0.207	(0.9)	0.0	-0.0	0.1	0.075	0.339
C. elegans	NA	NA	NA	NA	NA	NA	NA	NA	NA	NA

Names

- **catechol**
- 1,2-Dihydroxybenzene
- alpha-hydroxyphenol
- Brenzcatechin
- o-Benzenediol
- o-hydroxyphenol
- Pyrocatechol

Roles

genotoxin, allelochemical, plant metabolite

Status

Approved drug (according to ChEMBL)	No
Classification (according to DrugBank)	experimental
Number of Rule of 5 violations	0
Binding affinity to original target in log units (RF-Score prediction)	3.65
Burns *C. elegans* bioavailability prediction	1.51

Compound Target Characteristics

Peroxiredoxin-5, mitochondrial

Best gene implication in ageing for this target family came from gene Q960M4 annotated in UniProt release 2014_02. Annotation GO 8340 (determination of adult lifespan) was Inferred from Mutant Phenotype

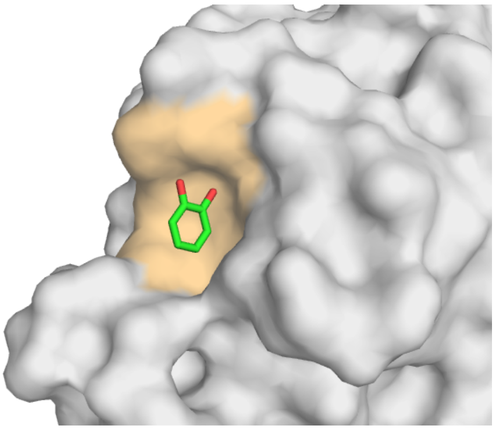

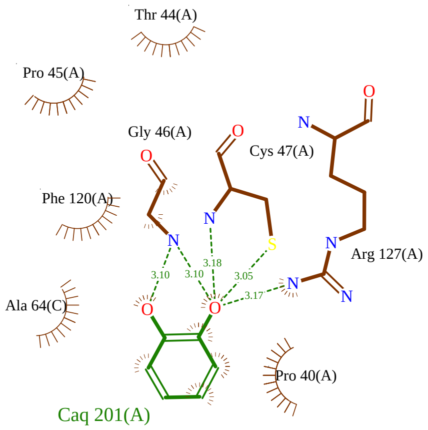

protein	amino acids contacts (binding site)
PDB:4k7i:chainA:P30044	P T P G C F R
sp:P30044:PRDX5_HUMAN	P T P G C F R
tr:D3ZEN5:D3ZEN5_RAT	P T P G C F R
sp:Q9R063:PRDX5_RAT	P T P G C F R
tr:G3UZJ4:G3UZJ4_MOUSE	P T P G C F R
tr:Q3UWS9:Q3UWS9_MOUSE	P T P G C F R
sp:P99029:PRDX5_MOUSE	P T P G C F R
tr:H3BJQ7:H3BJQ7_MOUSE	P T P G C F R
tr:Q960M4:Q960M4_DROME	P T P G C L R
sp:P38013:AHP1_YEAST	P S P T C D R

	whole protein		domain-based		contact-based	
protein	ident	simil	ident	simil	ident	simil
PDB:4k7i:chainA:P30044	0.99	1.0	0.99	1.0	1.0	1.0
sp:P30044:PRDX5_HUMAN	1.0	1.0	1.0	1.0	1.0	1.0
tr:D3ZEN5:D3ZEN5_RAT	0.66	0.73	0.84	0.93	1.0	1.0
sp:Q9R063:PRDX5_RAT	0.78	0.91	0.89	0.97	1.0	1.0
tr:G3UZJ4:G3UZJ4_MOUSE	0.59	0.7	0.66	0.72	1.0	1.0
tr:Q3UWS9:Q3UWS9_MOUSE	0.76	0.87	0.89	0.97	1.0	1.0
sp:P99029:PRDX5_MOUSE	0.77	0.9	0.89	0.97	1.0	1.0
tr:H3BJQ7:H3BJQ7_MOUSE	0.66	0.78	0.85	0.95	1.0	1.0
tr:Q960M4:Q960M4_DROME	0.46	0.73	0.57	0.84	0.86	0.97
sp:P38013:AHP1_YEAST	0.19	0.55	0.23	0.64	0.57	0.54

Prx5 (FBgn0038570) associated phenotypes

chemical resistant, chemical sensitive, immune response defective, increased cell death, long lived, parental effect, short lived, stress response defective

(Information from FlyBase)
